# Dynamic Task Offloading for Cloud-Assisted Vehicular Edge Computing Networks: A Non-Cooperative Game Theoretic Approach †

**DOI:** 10.3390/s22103678

**Published:** 2022-05-12

**Authors:** Md. Delowar Hossain, Tangina Sultana, Md. Alamgir Hossain, Md. Abu Layek, Md. Imtiaz Hossain, Phoo Pyae Sone, Ga-Won Lee, Eui-Nam Huh

**Affiliations:** Department of Computer Science and Engineering, Kyung Hee University, Global Campus, Yongin-si 17104, Korea; delowar@khu.ac.kr (M.D.H.); tangina@khu.ac.kr (T.S.); alamgir@khu.ac.kr (M.A.H.); layek@khu.ac.kr (M.A.L.); hossain.imtiaz@khu.ac.kr (M.I.H.); phoopyae@khu.ac.kr (P.P.S.); gawon@khu.ac.kr (G.-W.L.)

**Keywords:** vehicular edge computing, task offloading, multi-access edge computing, game theory

## Abstract

Vehicular edge computing (VEC) is one of the prominent ideas to enhance the computation and storage capabilities of vehicular networks (VNs) through task offloading. In VEC, the resource-constrained vehicles offload their computing tasks to the local road-side units (RSUs) for rapid computation. However, due to the high mobility of vehicles and the overloaded problem, VEC experiences a great deal of challenges when determining a location for processing the offloaded task in real time. As a result, this degrades the quality of vehicular performance. Therefore, to deal with these above-mentioned challenges, an efficient dynamic task offloading approach based on a non-cooperative game (NGTO) is proposed in this study. In the NGTO approach, each vehicle can make its own strategy on whether a task is offloaded to a multi-access edge computing (MEC) server or a cloud server to maximize its benefits. Our proposed strategy can dynamically adjust the task-offloading probability to acquire the maximum utility for each vehicle. However, we used a best response offloading strategy algorithm for the task-offloading game in order to achieve a unique and stable equilibrium. Numerous simulation experiments affirm that our proposed scheme fulfills the performance guarantees and can reduce the response time and task-failure rate by almost 47.6% and 54.6%, respectively, when compared with the local RSU computing (LRC) scheme. Moreover, the reduced rates are approximately 32.6% and 39.7%, respectively, when compared with a random offloading scheme, and approximately 26.5% and 28.4%, respectively, when compared with a collaborative offloading scheme.

## 1. Introduction

With the evolution of intelligent vehicles, the emergence of delay-sensitive and computation-intensive vehicular applications for things such as driving safety, intelligent navigation, autonomous driving, augmented vehicular reality, information entertainment, and accident warnings has increased rapidly [1,2,3,4]. The above applications are used to assist both drivers as well as passengers in vehicular networks. These applications usually have a large amount of processing data and require extensive resources during computation. However, the computational capabilities in vehicles are limited.

Therefore, it is difficult to ensure the requirements of the above-mentioned applications during computation, and still provide low latency and the required quality-of-service (QoS). To overcome the conflicts between resource-constrained vehicles and resource-intensive applications, cloud-based VNs were previously proposed as an effective approach, where a vehicle offloads its computation tasks to the resource-rich cloud infrastructure [5]. Offloading tasks to a remote cloud server greatly improves the computing performance and extends the computing capacity of the vehicle.

However, due to the long propagation delay between a vehicular user and cloud servers, unstable connections and unacceptable latency occurs, which reduces the offloading performance significantly. To tackle these challenges, a prominent approach has emerged, which is known as multi-access edge computing (MEC) [6]. MEC provides a new paradigm for fetching services in close proximity to the VNs. Thus, the vehicular user can obtain a faster response by offloading computing tasks to MEC-enabled networks. However, if the number of offloaded tasks from the vehicles to MEC server’s increases, it degrades the vehicular performance due to the overloading problem. Therefore, an efficient vehicular network is a crucial need to overcome the above challenges [7].

VEC has emerged as a promising technology where the vehicle can offload their computed tasks to local RSUs for swift computation and efficient storage [8]. At the edge of the VNs, it offers cloud-computing capabilities that handle real-time and computation-intensive tasks with low latency. VEC is basically the integration of traditional VNs with the emerging MEC. By using lightweight but ubiquitous MEC servers to extend the computation capacity of VNs poses significant challenges, particularly in dense traffic environments with a huge amount of demand from vehicles. This is due to vehicular mobility, the limited storage and resource capabilities in MEC servers. As the vehicular network environment is extremely dynamic, vehicles do not have enough information in advance about network conditions and edge resources, which therefore degrades performance. To deal with these challenges, most of the previous studies consider vehicle movement at a constant speed, or static vehicle positions when designing their VN models. Without considering vehicle movements at various speeds in VN models, it is challenging to apply in real life.

Moreover, VEC has suffered three major problems, which are the limited coverage ranges of RSUs, the overloaded problem, and the high mobility of vehicles. Furthermore, existing research ignored cloud–server resources during task offloading. On the other hand, it is not possible to predict the demand of the computing task generated by the vehicles in advance. Therefore, dynamic task offloading is an online problem that requires a solution with low-complexity.

To fill this gap and overcome the above-mentioned challenges, we designed an efficient dynamic task offloading approach for VNs based on a non-cooperative game. Game theory (GT) is a powerful tool for making decisions to offload tasks from among multiple offloading decisions, despite having limited resources. In a non-cooperative game, each vehicle can make its own strategy on whether a task is offloaded to a MEC server or a cloud server to maximize its benefits. Moreover, the advantages of using non-cooperative game-theory-based NGTO scheme uses a lightweight and distributed algorithm, which is an important criterion in online algorithms.

In addition, we consider a real-life scenario when designing the VN model by considering movement at various speeds. In our proposed scheme, each vehicle can select the optimal offloading decision, which reduces the task’s completion delay in each decision slot. This paper is an extension of our previous work [9]. We summarize the contribution of this paper as follows:

- We investigate the task-offloading problem in VNs to ensure the QoS and to accommodate a greater vehicular workload in MEC-enabled VNs.
- We propose an NGTO approach where vehicles can dynamically adjust the task-offloading probability to acquire the maximal utility. Moreover, we used a best response offloading strategy for deciding where the task will be offloaded.
- For local RSU computing, we use vehicle-to-RSU (V2R) communication mode and consider the movement of vehicles at various speeds when designing the VN model. Moreover, in our proposed model, vehicles are capable of offloading their computing tasks to a remote server in alternative ways—one via a base station (BS) and the other via RSUs.
- Finally, an extensive simulation analysis is conducted to demonstrate the efficiency of our proposed NGTO scheme, compared to its competitors, by reducing the task-failure rates and response times of infotainment, danger assessment, and navigation applications.

The rest of this paper is organized as follows. Section 2 describes related works, and summarizes the work on task offloading in VNs. In Section 3, we present a problem scenario for vehicular networks and our proposed system model. The basic game model and our proposed NGTO algorithm, including the offloading strategy for vehicles, a price function, and a utility function, are demonstrated in Section 4. Afterward, we discuss the performance evaluation of our proposal in Section 5. Lastly, in Section 6, we conclude this paper.

## 2. Related Work

Recently, task offloading in VNs has gained widespread attention in both industry and academia. A set of researchers are focused on optimizing the task-offloading problem from various perspectives [10,11,12,13,14,15,16,17,18]. To utilize the resources that are available in the nearby vehicles and to minimize the total latency, Wang et al. [10] introduced federated offloading of different vehicle-to-vehicle (V2V) and vehicle-to-infrastructure (V2I) communication in VNs. Based on the computation and communication environment, the computation task is processed in three ways: computed locally in the vehicle itself, offloading the computed task to neighboring vehicles through V2V communications, and offloading to a local MEC server through V2I communications. Moreover, systems adopt a distributed algorithm to use nearby vehicle resources for offloading tasks in V2V communications. In [11], the authors investigated the task-offloading problem for multi-user VNs, and proposed a load-balancing task-offloading solution. They developed an optimization algorithm to choose the target VEC server to maximize the system utility.

Xiao et al. [12] introduced a multi-agent reinforcement-learning-based task offloading strategy for the Internet of Vehicles (IoV). To balance between the task offloading delay and cost, they developed a three-stage Stackelberg game model. Moreover, Bozorgchenani et al. [13] proposed an online network selection scheme based on traffic patterns and congestion in vehicular edge networks using multi-armed bandit (MAB) theory to reduce the task offloading latency. By exploiting the historical offloading data set, they also developed an off-policy network selection approach to select the least congested network. To provide a computing service with low-latency, Cui et al. [14] proposed a double-deep Q-network (DDQN)-based cooperative vehicles-assisted task offloading strategy through V2V cooperation. They considered the computing resources that are available in the adjacent vehicles and used DDQN to determine the optimal task-offloading ratio.

In [15], a context-aware task offloading scheme was introduced based on software-defined network (SDN) technology for a collaborative VEC system. To solve the joint optimization problem of task offloading decision and resource allocation, they developed a differential evolution (DE) algorithm. Furthermore, to predict idle computing resources, an autoregressive integrated moving average (ARIMA) model was employed to cooperate vehicular task offloading. Karimi et al. [16] introduced an efficient task offloading scheme among MEC and central cloud in VNs based on deep-reinforcement learning (DRL). They utilized DRL to guarantee the required response time and automatically learn the dynamics of the network state. Moreover, a collaborative framework was analyzed to overcome the resource limitations of MEC-enabled networks [17,18].

Generally, the vehicular network environment is highly dynamic and uncertain. In static or slowly varying environments, traditional task offload methods may be useful; however, it is difficult to make the decision when the VNs are changing frequently. Currently, different studies are focused on the game theoretic approach, which is a strong tool for making task offloading decisions in VNs.

Zhang et al. [19] developed a hierarchical VEC offloading approach to meet vehicle demands on resource-constrained VEC servers by using a backup computing server in nearby places. They used a Stackelberg game to optimize the utility of each vehicle. However, they considered the movement of vehicles at a constant speed, which is challenging to apply in real life. Moreover, Zhang et al. [20] introduced predictive relay transmissions and a direct uploading scheme in a MEC-enabled VNs to reduce the transmission latency. However, their assumption is impractical due to their consideration of MEC servers as unlimited resources.

Zhou et al. [21] developed a novel big data–enabled energy-efficient VEC (BEGIN) framework for resource allocation based on a Stackelberg game. To make the decision on selecting the task offload server and for choosing a pricing strategy, Liwang et al. [22] introduced a Stackelberg-game-based V2V task offloading scheme. However, it is difficult to process latency-intensive tasks by considering only nearby vehicles (V2V) for task-offloading decisions.

A multi-user, non-cooperative game-based approach was proposed by Wang et al. [23] to determine each vehicle’s offloading probability by considering the communication overhead and QoS constraints. However, they considered only a single RSU for computation offloading and to avoid the handover problem. A sequential Stackelberg game approach was proposed by Ye et al. [24] for improving the performance in VEC servers, which optimized the strategy of workload allocation among resource-demand terminals, the VEC server, and resource-provision terminals.

To use the resources that are ideal in volunteer vehicles, Zeng et al. [25] proposed a volunteer-assisted VEC model. To analyze the interactions between VEC servers and the requesting vehicles, they used a Stackelberg game based on utility and cost functions. On the other hand, a context-aware, distributed task-offloading strategy was developed by using matching game theory in VEC [26]. This scheme reduces the average task-offloading delay as well as the energy consumption in edge nodes. However, the matching request between the RSUs and vehicle can create an additional overhead and slow down the offloading decision. Liu et al. [27] introduced a game-theory-based distributed algorithm for minimizing costs and reducing the offloading delay to compute tasks of vehicles in multi-user VNs. The different task offloading approaches in MEC-enabled VEC networks [19,20,21,22,23,24,25,26,27] based on game theory are summarized in Table 1.

## 3. Problem Scenario and System Model

In this section, we demonstrate the problem scenario in vehicular networks. Moreover, the architecture of our proposed system is illustrated in detail.

### 3.1. Problem Scenario

**Figure 1:**
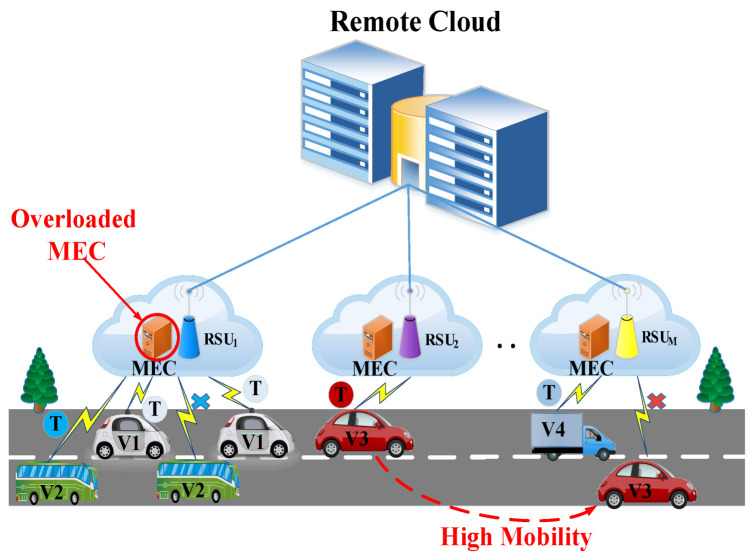
The problem of overloading and high mobility issues in vehicular networks.

The overloaded problem and high mobility in the vehicles are still challenging issues in dynamic multi-user VNs. When a huge number of vehicles offload their computing tasks to the same edge servers simultaneously, the processing delay is longer due to congestion. Thus, this degrades vehicular performance. Therefore, offloading the computing task to the closest edge server is not always the best decision. Figure 1 shows such scenarios. From this figure, we observe that, due to the high offloading requests, RSU${}_{1}$ is overloaded.

For example, $f_{i}^{mec}$ represents the maximum computational resource capacity of a MEC server, and $v\omega_{1},v\omega_{2},\ldots ,v\omega_{n}$ depicts the vehicle workload received by RSU${}_{i}$ from *N* vehicles, where $\left\{RSU_{i}|i=1,2,3,\cdots ,M\right\}$ and $\left\{v\omega_{i}|i=1,2,3,\cdots ,N\right\}$. Then, the entire workload received at RSU${}_{i}$ from *N* vehicles is:

(1) $$W_{i}^{mec}=\sum_{i=1}^{n}v\omega_{i}$$

Therefore, the rest of the computing capacity of MEC server, $\alpha_{i}$ can be calculated by using Equation (2).

(2) $$\alpha_{i}=f_{i}^{mec}-W_{i}^{mec}$$

When $\alpha_{i}<0$, the MEC will require additional resources for computing the task due to the overloaded problem. In this circumstance, it is difficult to decide whether the MEC server or the remote server be used to offload the computing task. Therefore, to handle the overloaded problem of RSU${}_{1}$ and to reduce the response time, we propose a game-theory-based efficient task offloading scheme. Based on our proposed game model, each vehicle decides where to offload computation tasks for processing, either in a MEC server or a cloud server.

On the other hand, high mobility in the vehicles is a major problem in VNs. Figure 1 shows some scenarios that are explained below.

- Scenario 1: Vehicles V1 and V2 enter the coverage area of $RSU_{1}$ and offload their task to the associated MEC server. The vehicle V1 task is processed successfully by that MEC server; however, vehicle V2 faces the overloaded problem for task execution.
- Scenario 2: Vehicle V3 is placed in the $RSU_{2}$ coverage area and offloads a task to the associated MEC server. Before finishing that task, the vehicle has already passed multiple RSU coverage areas. The vehicle enters the coverage area of $RSU_{M}$ when the task is finished.

From the above scenario, we can see that vehicle V1 does not face any problems during the task offloading and execution processes. On the other hand, vehicles V2 and V3 face certain problems during task execution and the reception of the results due to the overloaded and high-mobility problems. Therefore, in our proposed architecture, we use mobility-based task migration among the different MEC servers through a metropolitan area network (MAN).

When a vehicle moves to the coverage area of another RSU before executing the offloaded task in the original MEC server, our system forwards the obtained result from the original MEC server to the vehicle through the MAN and other RSUs in a multi-hop manner. However, our proposed system uses the approach in [28] to handle the handover problem by using the MAN.

### 3.2. Proposed System Model

**Figure 2:**
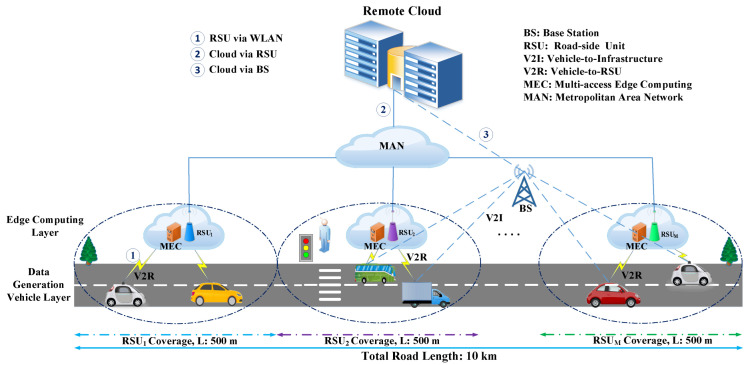
The proposed MEC-enabled vehicular network.

The proposed MEC-enabled vehicular network architecture is represented in Figure 2, which includes three layers: the vehicle layer, the edge-computing-network layer, and the remote cloud layer. The vehicles are located in the first layer of the architecture and use RSUs or a BS to establish a connection with the internet. The edge-computing-network layer contains RSUs and BS. For transmitting data between a MEC server and vehicles, vehicles use wireless communication techniques to communicate with the RSUs. Moreover, in our proposed architecture, we use a MAN to connect all the RSUs, sharing the computing resources via task migration.

Therefore, the edge computing layer can be considered as a shared resource pool. In our model, RSUs can also access remote cloud resources by using a fiber communications link. However, on the road, the BS is located such that it has a large coverage area so that vehicles can access it easily. Finally, in the third layer, the traditional remote cloud server is placed to provide powerful cloud computing services to vehicles. There are two ways to offload a vehicular computing task to the remote cloud under our proposed model: via the BS and via RSUs.

The proposed model consist of a set of N vehicles as N={1,2,3,⋯,N}. We assume each vehicle has generated *T* independent tasks when it arrives on the road, where $T=\{T_{i}|i=1,2,3,\cdots ,T\}$, and each computation task is characterized by, $T_{i}=\left\{d_{i}^{in},c_{i},t_{i}^{max}\right\}$. For task $T_{i}$, $d_{i}^{in}$ represents the size of input task; $c_{i}$ denotes the desired computing resource for task processing; and $t_{i}^{max}$ is the highest tolerable latency for $T_{i}$. Each task can be processed on the MEC server, offloaded to a cloud server by using an RSU, or accomplished on the cloud server through the BS.

Therefore, each vehicle has three offloading choices for accomplishing tasks. Moreover, a unidirectional road is considered in our proposed architecture. There are M RSUs with the same coverage area along the road, and they are deployed equidistantly. We represent the set of RSUs as $RSU=\{RSU_{1},RSU_{2},\cdots ,RSU_{M}\}$, where $\left\{RSU_{i}|i=1,2,\cdots ,M\right\}$. We divide the road into M segments of length L, where $\left\{L_{i}|i=1,2,\cdots ,M\right\}$, and all vehicles are randomly distributed in the segments of the road. $RSU_{m}$ can only be accessed for offloading tasks when the vehicles are located within the *m*th segment. Each RSU has a 500 m communication range. A single MEC server is equipped at each RSU (which has limited computing resources and storage capacity) to provide task offloading services for vehicles. For each MEC server, $MEC_{i}=\left\{f_{i}^{mec},s_{i}^{mec}\right\}$, where $f_{i}^{mec}$ and $s_{i}^{mec}$ represent the computing and storage capacity of $MEC_{i}$.

### 3.3. Communication and Computation Model

According to our proposed model, the offloaded task can be processed by using an edge server or remote cloud. The total task-completion time for both cases consists of the task transmission and processing time.

#### 3.3.1. Edge Offloading

When vehicle *i* chooses a MEC server that is connected to $RSU_{m}$ for offloading computation task $T_{i}$, the total processing time can be computed as follows:

(3) $$t_{i,m}^{mec}=v_{i,m}^{up}+v_{i,m}^{exe}+v_{i,m}^{d}$$

where $v_{i,m}^{up}$ represents the uplink delay during transmission, while the vehicle *i* offloads its computing task to the $RSU_{m}$ connected to the MEC server, and $v_{i,m}^{d}$ depicts the downlink delay during transmission, while the *i*th vehicle receives the results. Moreover, $v_{i,m}^{exe}$ denotes the execution time for processing vehicle *i*’s task on the MEC server, which is derived as follows:

(4) $$v_{i,m}^{exe}=\frac{d_{i}^{in}}{f_{m}^{mec}}\left(1-U_{m}^{mec}\right)$$

Here, $d_{i}^{in}$ is the task size, while $f_{m}^{mec}$ and $U_{m}^{mec}$ are the computing capability and the utilization of the $RSU_{m}$ connected to the MEC server, respectively.

#### 3.3.2. Cloud Offloading

In our proposed model, vehicles have two ways to offload and compute their tasks at a remote server. When vehicles use an RSU for offloading their task to a remote server, the total processing time of vehicle *i*’s task can be obtained by:

(5) $$t_{i,rsu}^{cloud}=v_{i,crsu}^{up}+v_{i,c}^{exe}+v_{i,crsu}^{d}$$

where $v_{i,crsu}^{up}$ and $v_{i,crsu}^{d}$ are the uplink and downlink delay, respectively, via the RSU. Moreover, $v_{i,c}^{exe}$ represents the processing time to execute the task in the cloud. However, when the task is offloaded to a remote server from vehicle *i* through the BS, the total processing time of vehicle *i*’s task can be obtained by:

(6) $$t_{i,bs}^{cloud}=v_{i,cbs}^{up}+v_{i,c}^{exe}+v_{i,cbs}^{d}$$

where $v_{i,cbs}^{up}$ and $v_{i,cbs}^{d}$ are the uplink and downlink delay, respectively, via the BS. Moreover, $v_{i,c}^{exe}$ represents the task-execution time in the cloud. In both cases (task offloading via RSU or BS), execution time $v_{i,c}^{exe}$ is the same and can be calculated as follows:

(7) $$v_{i,c}^{exe}=\frac{d_{i}^{in}}{f^{cloud}}\left(1-U^{cloud}\right)$$

Here, $U^{cloud}$ and $f^{cloud}$ represent the average utilization and computation capability of the cloud server, respectively.

## 4. The Non-Cooperative Game Theory-Based Task Offloading Algorithm

In this section, we first present the task-offloading-game model. Afterward, we discuss our proposed NGTO algorithm.

### 4.1. The Basic Game Model

Game theory is a powerful tool with low complexity in developing distributed algorithms. It can make a task offloading decision where the vehicle can achieve maximum utility. The basic task offloading strategy of the game can be represented by using a triplet [29], $G=\{N,{\left(p_{i}\right)}_{i\in N},{\left(u_{i}\right)}_{i\in N}\}$, where

- N={1,2,3,⋯,n},i∈N is a set of *N* finite players (vehicles)
- ${\left(p_{i}\right)}_{i\in N}$ is a set of offloading strategies for vehicle *i*, and the possible strategy of vehicle *i* is any of $p_{i}$, where $p_{i}=\left\{p_{i}\in \left\{p_{i}^{mrsu},p_{i}^{crsu},p_{i}^{cbs}\right\},p_{i}^{s}\in \left\{0,1\right\},i\in N,s\in \left\{mrsu,crsu,cbs\right\}\right\}$. Here, {mrsu,crsu,cbs} denotes the offloading decision set, where mrsu represents a task that offloads to a MEC server that is connected to RSU, crsu denotes task offloads to a cloud server via RSUs, and cbs indicates task offloads to a cloud server via the BS. Moreover, $p_{i}=p_{i}^{s}=1$ indicates that vehicle *i* has completed its task by choosing decision *s*; otherwise $p_{i}=p_{i}^{s}=0$.
- ${\left(u_{i}\right)}_{i\in N}$ is the payoff (utility) function of vehicle *i*, which can be represented as $u_{i}\left(p\right)$. Each vehicle is attempting to find out the strategy that is more profitable when offloading the task in order to maximize its utility, i.e.,
  (8) $$\begin{array}{c} p_{i}=\max_{p_{i}}\;u_{i}\left(p\right) \end{array}$$

### 4.2. Payoff Function

Each player in the game tries to maximize the global payoff. The payoff function can be defined as follows:

(9) $$u_{i}\left(p\right)=A_{i}\left(p\right)-R_{i}\left(p\right)$$

where $A_{i}\left(p\right)$ and $R_{i}\left(p\right)$ depict the utility and price functions, respectively. We employ the task execution time to evaluate the utility for the vehicle. The vehicle will achieve a higher utility when the task is accomplished before the tolerable latency; on the other hand, the vehicle will not receive that benefit if the task execution time goes past the tolerable latency. Therefore, according to the above-mentioned principle, the utility function for offloading the task of vehicle *i* is derived as follows:

(10) $$A_{i}\left(p\right)=\frac{t_{i}^{max}-t_{i,m}^{mec}}{t_{i}^{max}}\left(1-p_{i}\right)+\frac{t_{i}^{max}-t_{i}^{cloud}}{t_{i}^{max}}p_{i}$$

where $\frac{t_{i}^{max}-t_{i,m}^{mec}}{t_{i}^{max}}$ and $\frac{t_{i}^{max}-t_{i}^{cloud}}{t_{i}^{max}}$ represent the utility from vehicle *i*’s task being accomplished by the MEC server or the cloud server, respectively. The vehicle will obtain a negative utility, according to Equation (10), if the task execution time exceeds $t_{i}^{max}$.

The MEC server consists of limited computing and storage resources. Moreover, the remote cloud has sufficient computing resources. Therefore, in our proposed system, we used a dynamic-pricing strategy to control the task-offloading behavior of the vehicle. Based on the strategy of the dynamic-pricing mechanism used in [30], the price function can be derived as follows:

(11) $$R_{i}\left(p\right)=p_{i}^{2}\rho \left[\left(1-\prod_{j\neq i}\left(1-\iota_{j}p_{j}\right)\right)\right]$$

where ρ∈[0,1] represents the pricing factor that determine where the task will be offloaded, and ι depicts the task arrival rate. If the MEC server has sufficient storage and computing resources, then we use a low pricing factor to ensure efficient use of the MEC resources. Therefore, by substituting the values of $A_{i}\left(p\right)$ and $R_{i}\left(p\right)$ from Equations (10) and (11) into Equation (9), the payoff function can be expressed as follows:

(12) $$\begin{array}{c} u_{i}\left(p\right)=\frac{t_{i}^{max}-t_{i,m}^{mec}}{t_{i}^{max}}\left(1-p_{i}\right)+\frac{t_{i}^{max}-t_{i}^{cloud}}{t_{i}^{max}}p_{i}\\ -p_{i}^{2}\rho \left[\left(1-\prod_{j\neq i}\left(1-\iota_{j}p_{j}\right)\right)\right] \end{array}$$

### 4.3. Proposed Algorithm

We use the NGTO-based dynamic task offloading scheme for VNs in this study where each vehicle can make its own strategy on whether a task is offloaded to a MEC server or a cloud server to maximize its benefits. Moreover, in our proposed model, there are two ways for offloading a vehicular computing task to the remote cloud: via the RSUs and via BS. We use Algorithm 1 for our proposed NGTO approach, where each vehicle can dynamically adjust the task-offloading probability to acquire the maximal utility. For all tasks, vehicles can make a pre-selection in the cloud-computing processing model from the RSUs or BS.
**Algorithm 1:** NGTO algorithm.1:Definitions: crsu (cloud offloading via RSU), cbs (cloud offloading via BS).2:Initialization: vehicle index *i*, task arrival rate ι, pricing factor ρ, offloading probability *p*.3:**for all** task t∈T **d**o4:     Estimate uplink transmission delay $v_{i,crsu}^{up}$ and downlink transmission delay $v_{i,crsu}^{d}$     of $t^{th}$ task for WAN // Cloud offloading via RSU5:     Estimate uplink transmission delay $v_{i,cbs}^{up}$ and downlink transmission delay $v_{i,cbs}^{d}$     of $t^{th}$ task for WAN over LTE // Cloud offloading via BS6:     $v_{i,c}^{exe}=\frac{d_{i}^{in}}{f^{cloud}}\left(1-U^{cloud}\right)$ // Task processing time in the cloud7:     $t_{i,rsu}^{cloud}=v_{i,crsu}^{up}+v_{i,c}^{exe}+v_{i,crsu}^{d}$ // Calculate total completion time of task in cloud     via RSU8:     $t_{i,bs}^{cloud}=v_{i,cbs}^{up}+v_{i,c}^{exe}+v_{i,cbs}^{d}$ // Calculate total completion time of task in cloud via BS9:     Compare $t_{i,rsu}^{cloud}$ and $t_{i,bs}^{cloud}$, and select the minimum between them10:       **if** $t_{i,rsu}^{cloud}$>$t_{i,bs}^{cloud}$ **then**11:          $t_{i}^{cloud}=t_{i,bs}^{cloud}$ // Cloud offloading via BS12:       **else**13:          $t_{i}^{cloud}=t_{i,rsu}^{cloud}$ // Cloud offloading via RSU14:       **end if**15:     Estimate uplink transmission delay $v_{i,m}^{up}$ and downlink transmission delay $v_{i,m}^{d}$     of $t^{th}$ task for MEC offload16:     $v_{i,m}^{exe}=\frac{d_{i}^{in}}{f_{m}^{mec}}\left(1-U_{m}^{mec}\right)$ // Task processing time in the MEC server17:    $t_{i,m}^{mec}=v_{i,m}^{up}+v_{i,m}^{exe}+v_{i,m}^{d}$ // Calculate total task-completion time on MEC server18:     Update best-response offload strategy:    $p_{i}={\left[\frac{t_{i,m}^{mec}-t_{i}^{cloud}}{2\rho t_{i}^{max}\left(1-\prod_{j\neq i}\left(1-\iota_{j}p_{j}\right)\right)}\right]}_{0}^{1}$19:**end for**20:Vehicle *i* decides whether to offload task with probability $p_{i}$.

From lines 3 to 8, the total completion time of task in cloud via the RSU and the BS are calculated. To select the best one, we compared between the cloud offloading via RSU and cloud offloading via BS, shown in lines 9 to 14. From lines 15 to 16, the task-transmission delay and processing time are estimated in the MEC server. In line 17, we calculate the total task-completion time on the MEC server. However, we use a best response offloading strategy for the task-offloading game in order to achieve a unique and stable equilibrium. The updated best response offloading strategy of vehicle *i* can be expressed as follows:

(13) $$p_{i}=\max_{p_{i}}\;u_{i}\left(p\right)={\left[\frac{t_{i,m}^{mec}-t_{i}^{cloud}}{2\rho t_{i}^{max}\left(1-\prod_{j\neq i}\left(1-\iota_{j}p_{j}\right)\right)}\right]}_{0}^{1}$$

Then, each vehicle updates the offloading strategy based on Equation (13) and selects the most optimal offloading strategy to complete the vehicle offloaded task. According to [29,31], it is proven that Equation (13) is a contraction mapping, which than implies the game has a unique and stable NE. Therefore, the task-offloading game uses the best response strategy in Algorithm 1 that converges to a unique NE. Moreover, the operator ${\left[x\right]}_{0}^{1}$ can cause the probability $p_{i}\in \left[0,1\right]$ [30].

**Lemma** **1.** *In the game, there is a unique Nash equilibrium, if the best response mapping on the entire strategy space is a contraction [29].*

**Theorem** **1.** *The best response offloading strategy used in Algorithm 1 for the task-offloading game can converge to the unique NE, if*

(14) $$\sum_{j\neq i}\frac{|t_{i,m}^{mec}-t_{i}^{cloud}|\prod_{k\neq i,j}\left(1-\iota_{k}p_{k}\right)}{2\rho t_{i}^{max}{\left(1-\prod_{j\neq i}\left(1-\iota_{j}p_{j}\right)\right)}^{2}}<1\;\forall i\in \left\{1,\ldots ,n\right\}.$$

**Proof.**  See Appendix A. □

## 5. Performance Evaluation

In order to assess the performance of our proposed NGTO scheme based on different scenarios via EdgeCoudSim simulator [32], we compared our proposal with three benchmark offloading schemes that are explained as follows.

- Local RSU Computing (LRC): In this scheme, the vehicle’s computation tasks are offloaded for processing in a MEC server that is located nearby.
- Random Offloading: In the random scheme, vehicles randomly choose as the target server a MEC server or a remote server to process the offloaded task. Moreover, the probability of choosing any of the target servers is the same.
- Collaborative Offloading: In this scheme, some offloaded tasks from vehicles will be processed by a local RSU, while the rest will be offloaded and processed by a remote cloud through RSU. In collaborative offloading scheme, it is preferred to use local RSU to offload delay-sensitive smaller tasks whereas remote cloud server is used for processing delay-tolerant larger tasks.

### 5.1. Simulation Setup

**Figure 3:**
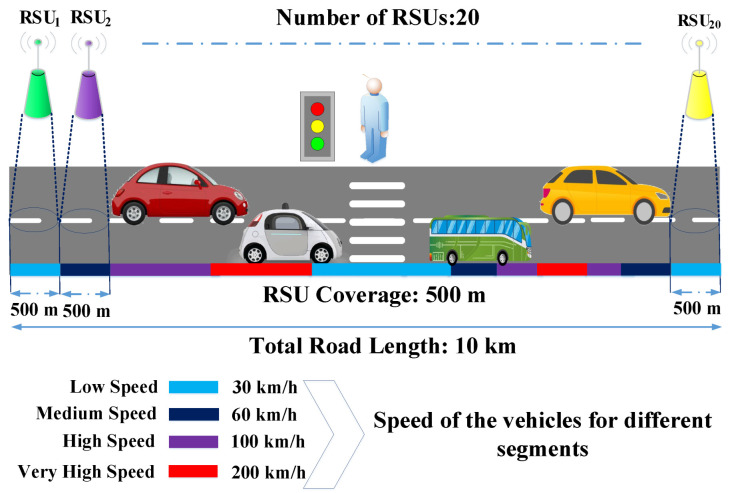
Vehicle movement mode for the simulation.

During the simulation, we envisage a 10 km unidirectional highway that is shown in Figure 3. For a more realistic simulation, the road is divided into segments. Moreover, we deployed 20 equidistant RSUs along the road, where RSU${}_{1}$ and RSU${}_{20}$ are located at the far left and far right of the road segments, respectively. We assume that from the far left side (the coverage area of RSU${}_{1}$) of the road, all the vehicles will enter. Moreover, we considered four different vehicular speeds (low, medium, high, and very high) at 30, 60, 100, and 200 km/h, respectively, during simulation. Generally, the computed task of vehicles are offloaded into a nearby RSU.

However, we assume that each RSU has an IEEE 802.11p–capable access point and a 500 m coverage area. A single MEC server with one host is equipped in each RSU, which operates four virtual machines (VMs). In this work, we use the Markov modulated process (MMPP/M/1 queue model) for the network delay model. In addition to that, for transmitting the computed task in case of V2R communication, we used IEEE 802.11p protocol interface with 10 Mbps data rate [33]. On the other hand, our system can offload a vehicle’s task through an RSU or an LTE base station to a cloud server. For the LTE BS, we used according to Xu et al.’s measurements [34] during the simulation analysis.

Additionally, each vehicle runs a danger assessment (DA), an infotainment (I), and a navigation application (NA) to produce various tasks during the experiments. Among them, the infotainment application is latency-tolerant, while the danger assessment and navigation applications are latency-sensitive.

The simulation parameters and the respective values are represented in Table 2 and Table 3 lists the key characteristics of three different applications used during the simulation [32,35]. In Table 3, usage represents the percentage of vehicles that request the related services for NA, DAA, and I applications. The inter-arrival time for the tasks depicts the frequency of generating tasks, which follows an exponential distribution. We considered three, five, and fifteen seconds as representing the inter-arrival times of the NA, DAA, and I applications, respectively.

For example, the navigation application generates smaller tasks every three seconds, and the infotainment application generates a bigger task every fifteen seconds. To differentiate the sensitivity of the various applications (latency-sensitive or latency-tolerant), we employed a latency-sensitivity value in our simulation. We used lower and higher values to represent the delay sensitivity in latency-tolerant and latency-sensitive applications, respectively. Hence, we set 0.25 for the delay sensitivity of the infotainment applications, and 0.8 for the danger assessment applications. Moreover, the other characteristics, such as the task length, maximum delay requirement and the upload and download data sizes of different applications, are given in Table 3.

### 5.2. Simulation Results

**Figure 4:**
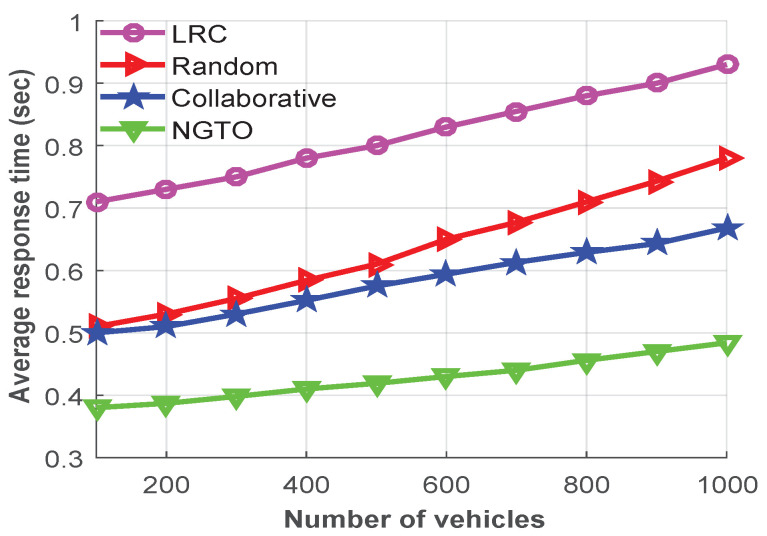
The average response time based on the number of vehicles.

To analyze the performance of the NGTO approach, Figure 4 represents the average response times (the y-axis) versus the numbers of vehicles (the x-axis, varying from 100 to 1000). From the figure, we can see that, with increasing number of vehicles, the average response time of all schemes also increases, and the LRC scheme has a higher response time than the others due to the congestion. When it comes to the other three approaches, the tasks will be distributed between the edge and the remote cloud.

Therefore, compared with LRC scheme, the response time does not increase for handling the large number of vehicles. However, due to the different distances between vehicle and RSU access point, the competitor schemes cannot keep the required latency at a stable level. The simulation results confirmed that the proposed NGTO approach has lower response time compared to others due to adjusting the task-offloading probability dynamically to maximize the utility of vehicles. Hence, the average response time will reduce by using our proposed approach at approximately 47.6%, 32.6%, and 26.5% from the LRC, random, and collaborative offloading approaches, respectively.

**Figure 5:**
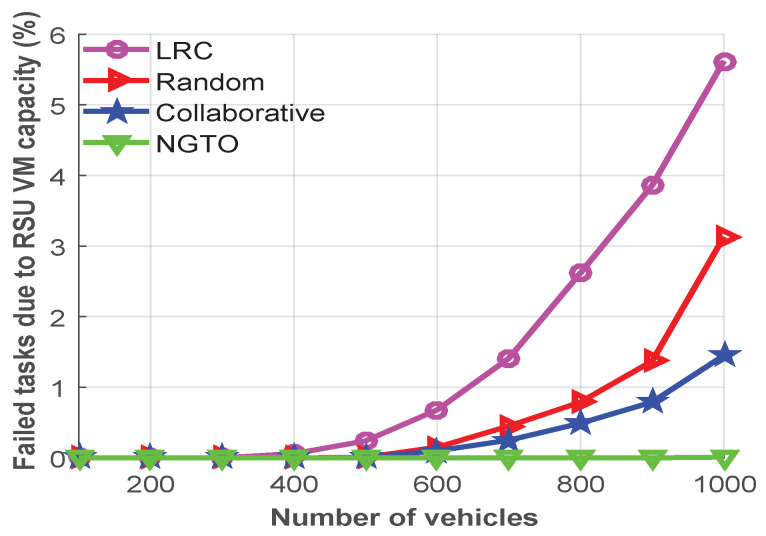
The average failed task rates due to the RSU VM capacity.

One of the most crucial performance criteria of VNs is the average unsuccessful task ratio. If the utilization of virtual machine is too high, the tasks are hard to handle and will fail more often. Figure 5 shows the task-failure rate due to the RSU VM capacity based on the number of vehicles. In each MEC server, we considered that there are four VMs during this experiment. From the figure, one can easily observe that in all offloading schemes, the task-failure rate is low until 500 vehicles. After that, the task-failure rate is increased due to congestion.

From Figure 5, we see that the LRC scheme starts to experience congestion after 400 vehicles, while the random and collaborative offloading schemes become congested after 600 vehicles; however without any congestion, the NGTO scheme can handle 1000 vehicles. After 400 vehicles, the LRC approach has to deal with the overloaded problem due to the restricted computing capacity of MEC server, and it starts to congest. However, by distributing the tasks between the edge and the cloud, the random and collaborative offloading schemes can easily handle 600 vehicles without congestion. After that, due to the WAN delay, they face congestion. Moreover, in the random offloading scheme, some larger tasks are sent to the MEC servers.

Therefore, the maximum response time allowed to process for these tasks is exceeded. On the other hand, our proposed NGTO approach is more capable for utilizing the resources of the MEC server than its competitors and can make dynamic decisions to offload tasks between a MEC server and the cloud. In addition to that, NGTO scheme attempts to converge to a unique and stable equilibrium. Therefore, it can easily handle 1000 vehicles without experiencing any congestion.

**Figure 6:**
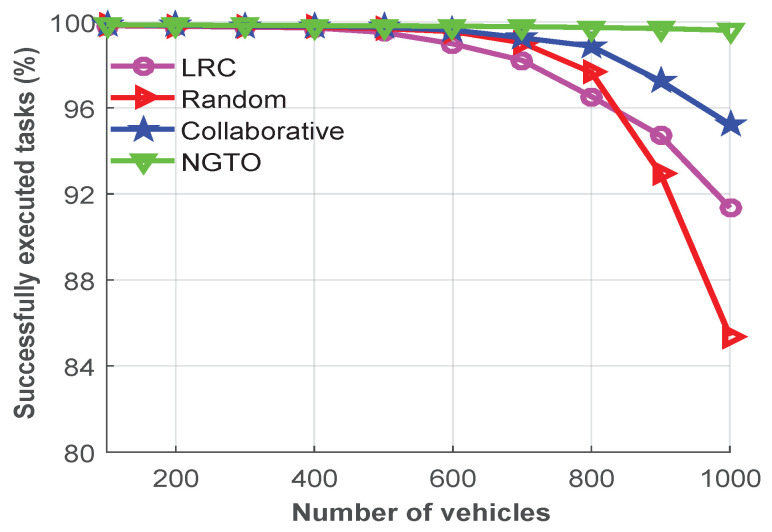
Successfully executed tasks for various numbers of vehicles.

Moreover, to measure the importance of our proposed scheme, Figure 6 demonstrates the successfully executed task rates for various numbers of vehicles. The successfully completed task rate indicates the percentage of tasks successfully executed out of the total number of tasks. From the figure, we observe that most of the offloaded tasks are executed successfully in all the schemes when the system is lightly loaded. On the other hand, if the number of vehicles increases, the situation will change. For instance, due to the limited capacity and overload problem at the MEC server in the LRC scheme, the number of successfully executed tasks will rapidly dropped from 99.5% at 500 devices to less than 91.4% at 1000 devices. However, in the random offloading approach, the probability of choosing all the target servers is the same, and it selects the offloading target server randomly. Therefore, the number of successfully executed tasks saw a huge drop in the random offloading scheme, from 99.6% at 500 devices to 85.4% at 1000 devices.

However, due to the WAN delay, the collaborative offloading scheme dropped from 99.7% at 500 devices to 95.2% at 1000 devices, whereas the NGTO approach showed a lower drop to only 99.5% at 1000 devices from 99.8% at 500 devices. Therefore, after analyzing Figure 6, we can summarize that our proposed NGTO approach is capable of executing successfully more offloaded tasks compared to the others. As our proposed scheme efficiently utilize the MEC resources and make better decisions for maximizing the utility when a vehicle forwards the task to the MEC server or to the cloud server.

**Figure 7:**
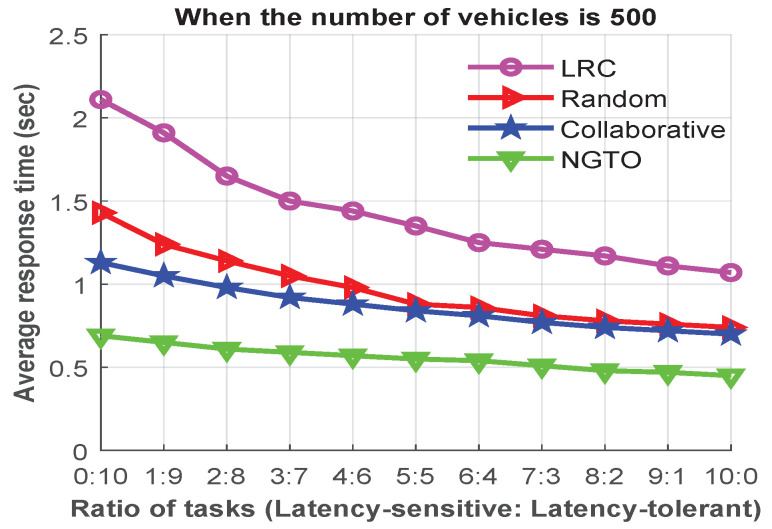
The average response time in terms of various ratios of latency-sensitive to latency-tolerant tasks.

By varying the ratio between the latency-sensitive danger assessment applications and the latency-tolerant infotainment applications, we performed another experiment to analyze the average response time with results shown in Figure 7. We used ratios from 0:10 up to 10:0 to investigate the effect of the ratios between these applications. For example, the ratio 7:3 indicates seven DA applications to three IA applications. At first, we assumed that all offloaded tasks are latency-tolerant (the 0:10 ratio). Then, from Figure 7, we see that the average response times of the LRC, random, collaborative, and NGTO schemes were 2.11, 1.43, 1.13, and 0.69 s, respectively.

The main reason for the higher response times in all offloading schemes is the larger task size for infotainment applications. However, when we decreased the number of latency-tolerant tasks compared to latency-sensitive tasks, the average response time also decreased. The task-completion time was almost stable in all offloading schemes when the ratio was between 6:4 and 10:0. However, in all scenarios, our proposed NGTO scheme outperformed in terms of reducing the response time, as the response time of our proposed scheme did not exceed the deadline of the task and was able to be sustained at a low value under different ratios of tasks.

**Figure 8:**
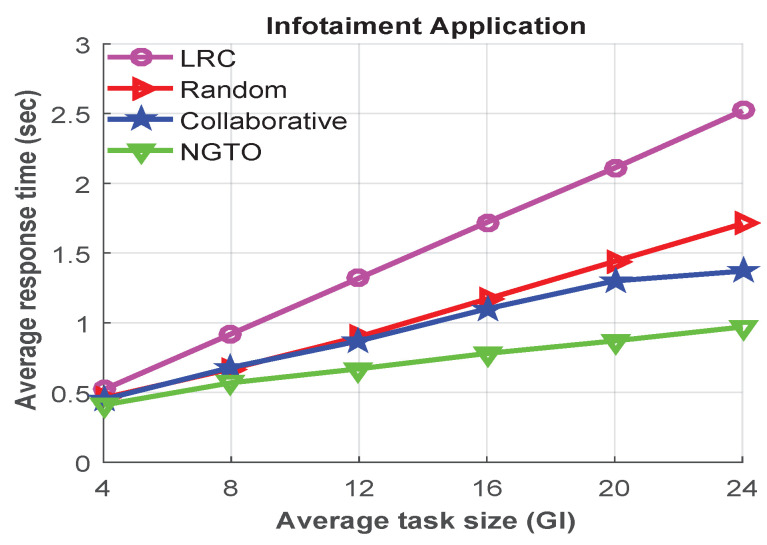
The average response times for different task sizes of infotainment applications.

**Figure 9:**
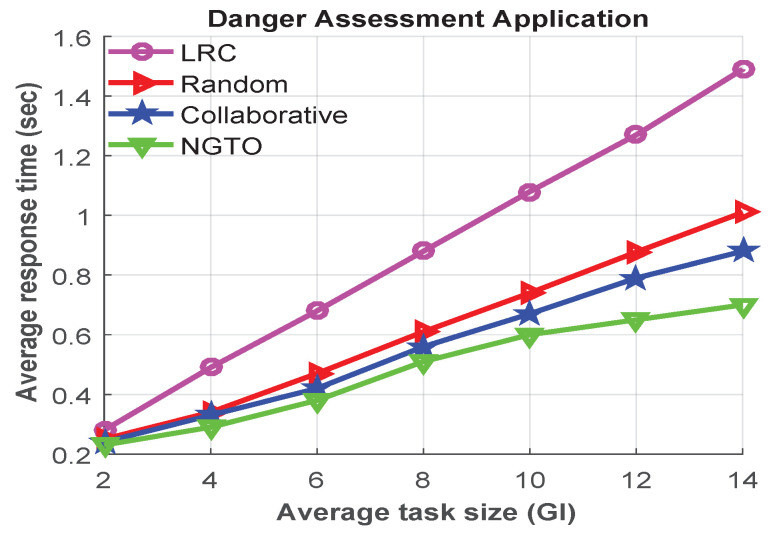
The average response times for different task sizes of danger assessment applications.

Moreover, we conducted other experiments to investigate the impacts of different task sizes of infotainment and danger assessment applications, which are shown in Figure 8 and Figure 9, respectively. From analyzing Figure 8 and Figure 9, we observed that, when the average task size is small, the average response time is almost similar in all offloading scheme. However, when the task size becomes larger, the response time is also increased.

However, the average response time is higher in the infotainment application compared to the navigation application. As the navigation application generates smaller tasks compared to the infotainment application. Through the above analysis, we conclude that our proposal outperformed the others in all scenarios. Because of our proposed NGTO scheme can converge to a unique equilibrium and make a dynamic decision for processing the offloaded task.

**Figure 10:**
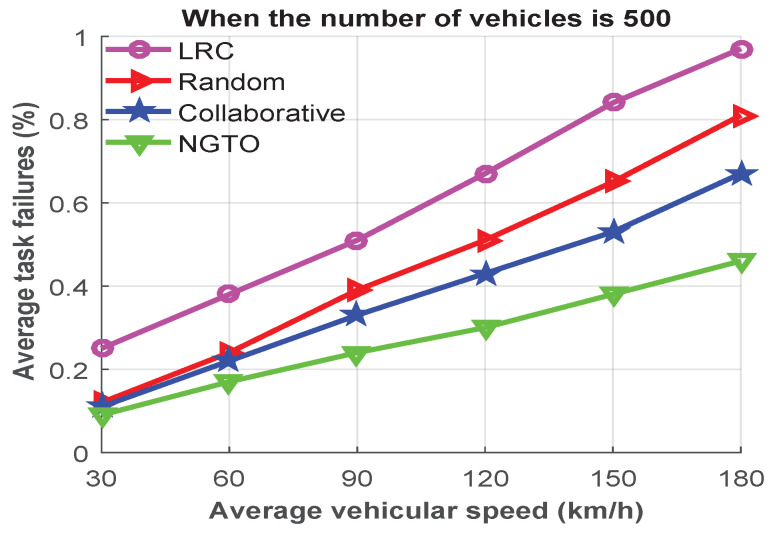
The average task-failure rates for different vehicular speeds.

Finally, the last simulation result in Figure 10 investigates the effect of various speed of vehicle in order to analyze the rate of the failed tasks. During the experiment, we used 500 vehicles and considered average speeds from 30 to 180 km/h. From analyzing Figure 10, we observe that, when the average speed was up to 60 km/h, the average task-failure rate was low and almost the same in all offloading schemes except for the LRC scheme. However, when the average speed increased, the situation became worse due to the high task-failure rate.

For instance, if the average vehicle speed was 120 km/h, the rates of failed tasks of the LRC, random, collaborative, and NGTO approaches were approximately 0.67%, 0.51%, 0.43%, and 0.3%, respectively. Through the above evaluation, we summarize that our introduced NGTO approach outperformed the others in all scenarios. It reduced task-failure rates by approximately 54.6%, 39.7%, and 28.4% corresponding to the LRC, random, and collaborative approaches, respectively.

## 6. Conclusions

Vehicular edge computing is a promising technology for meeting the demands of resource-constraint vehicles through task offloading. In this paper, we investigate the task-offloading problem in VNs, and propose an efficient dynamic task offloading approach based on a non-cooperative game. Our proposed strategy can dynamically adjust the task-offloading probability between the MEC server and the cloud to acquire the maximal utility for each vehicle. To compare our proposed strategy with other approaches in this study, we conducted experiments on different scenarios in the EdgeCloudSim simulator. To assess our proposed NGTO approach, we analyzed our proposed scheme in terms of three different applications, including infotainment, danger assessment, and navigation applications, to produce various tasks in the network.

Furthermore, we compare the performance of our proposal with three benchmark task-offloading schemes. In this study, we showed that our proposed best response offloading strategy that was used in the task offloading game could converge to a unique NE. Therefore, our proposal can largely enhance system performance and outperformed in all scenarios compared with the LRC, random, and collaborative offloading schemes in terms of reducing the task-failure rate and response time. Our proposed NGTO approach was capable of executing more offloaded tasks successfully compared to the others.

With the rapid development of VEC technology and the evolution of intelligent vehicles, a huge amount of data will be generated due to deploying computation-intensive applications in vehicles. Therefore, in the future, we will consider a machine-learning-based approach for an efficient task offloading strategy in MEC-enabled VEC networks. Moreover, we will expand our study where vehicles can choose to offload their tasks to nearby MECs or V2V offloading to maximize their utility.

## Figures and Tables

**Table 1:** Summary of the different game theory approaches for task offloading in MEC-enabled VEC networks.

Ref.	Proposed Algorithm	# of	Server	Objectives	W.H.	Scenario	V2V	V2R	V2I
		Vehicles						(Edge)	(Cloud)
Ref. [19]	Stackelberg game	120	Edge	RT	Single	Highway	×		×
Ref. [20]	Non-cooperative game	ND	Edge	RT	Multi	Highway			×
Ref. [21]	Stackelberg game	10	Edge	E	Single	Urban	×		×
Ref. [22]	Stackelberg game	ND	VC	RT	Single	Highway		×	×
Ref. [23]	Non-cooperative game	70	Edge	RT	Single	Highway	×		×
Ref. [24]	Sequential game	ND	Edge	RT	Single	No Data	×		×
Ref. [25]	Stackelberg game	90	VC, Edge	OC	Single	Highway			×
Ref. [26]	Matching game	100	VC, Edge	RT, E	Single	Highway			×
Ref. [27]	Potential game	30	Edge	RT	Single	No Data	×		×
Our Study	Non-cooperative game	1000	Edge, TC	RT	Sing., Mul.	Highway	×		

VC = Vehicular cloud, TC = Traditional cloud, ND = No data, RT = Response time, E = Energy, OC = Offloading cost, W.H. = Wireless hops.

**Table 2:** Simulation parameters.

Parameters	Value
Number of RSUs	20
Number of vehicles	100∼1000
Network delay model	MMPP/M/1 queue model
Number of VMs per MEC server	4
Number of VMs per Cloud	20
VM processing speed per MEC server	10 GIPS
VM processing speed in the Cloud	75 GIPS
RSU coverage radious	500 m
WLAN/MAN bandwidth	10/1000 Mbps
WAN/WAN over LTE bandwidth	50/20 Mbps
WAN/WAN over LTE propagation delay	150/160 ms

**Table 3:** Simulation parameters for the three types of applications.

Parameters	Application Types		
	Navigation	Danger Assessment	Infotainment
	Application (NA)	Application (DAA)	Application (IA)
Usage (%)	30	35	35
Inter-arrival time of tasks (s)	3	5	15
Delay sensitivity	0.5	0.8	0.25
Maximum delay requirement (s)	0.5	1	1.5
Upload data volume (KB)	20	40	20
Download data volume (KB)	20	20	80
Average length of task (GI)	3	10	20

## Data Availability

Not applicable.

## Appendix A. Proof of Theorem 1

We must prove that Equation (13) is a contraction mapping based on the contraction mapping theorem proposed by Abraham et al. [36] and Lemma 1 [29] by validating the Jacobian Matrix ${\left||J|\right|}_{\infty }<1$. The contraction mapping is the most frequently and widely used method that can easily verify that the best response mapping is a contraction with a unique fixed point. According to the best response strategy of Equation (13), the Jacobian matrix is constructed as follows:

(A1) $$J_{ij}=\frac{\partial p_{i}}{\partial p_{j}}=\left\{\begin{array}{c} 0,\;i=j\\ \frac{-\left(t_{i,m}^{mec}-t_{i}^{cloud}\right)\prod_{k\neq i,j}\left(1-\iota_{k}p_{k}\right)}{2\rho t_{i}^{max}{\left(1-\prod_{j\neq i}\left(1-\iota_{j}p_{j}\right)\right)}^{2}},\;otherwise. \end{array}\right.$$

It then follows that

(A2) $${\left||J|\right|}_{\infty }=\sum_{i\in \{1,\ldots .,n\},j\neq i}\frac{|t_{i,m}^{mec}-t_{i}^{cloud}|\prod_{k\neq i,j}\left(1-\iota_{k}p_{k}\right)}{2\rho t_{i}^{max}{\left(1-\prod_{j\neq i}\left(1-\iota_{j}p_{j}\right)\right)}^{2}}.$$

In the off-diagonal elements in *J*, the maximum value is identified after measuring the sum of absolute values, which is represented as ${\left||J|\right|}_{\infty }$ in (A2). Hence, $\sum_{j\neq i}\frac{|t_{i,m}^{mec}-t_{i}^{cloud}|\prod_{k\neq i,j}\left(1-\iota_{k}p_{k}\right)}{2\rho t_{i}^{max}{\left(1-\prod_{j\neq i}\left(1-\iota_{j}p_{j}\right)\right)}^{2}}<1.$ Therefore, we conclude that ${\left||J|\right|}_{\infty }<1$ according to the assumption in the theorem. Thus, Equation (13) is a contraction mapping that guarantees that the best response strategy that is used in Algorithm 1 is the unique NE.
